# Will the endophytic fungus *Phomopsis liquidambari* increase N-mineralization in maize soil?

**DOI:** 10.1371/journal.pone.0293281

**Published:** 2023-11-13

**Authors:** Nariman A. Elsharif, Madiha W. El awamie, Naeema Matuoog

**Affiliations:** 1 Faculty of Arts and Sciences, Department of Botany, University of Benghazi, Ghemins, Libya; 2 Faculty of Sciences, Department of Microbiology, University of Benghazi, Benghazi, Libya; Universitat Jaume 1, SPAIN

## Abstract

Endophytes can be developed into biocontrol agents and can be fungi, bacteria, or archaea that live inside plant tissues without causing symptoms of disease. *Phomopsis liquidambari* is an endophytic fungus that plays an important ecosystem role as a biofertilizer by helping its host obtain soil nitrogen. How this fungus impacts N mineralization and microbial communities is little known. Our understanding of soil nutrient transformations and soil-plant-microbe interactions in *Phomopsis liquidambari*-crop versus conventional crop systems is incomplete. This study provided a better understanding of the effect of *Phomopsis liquidambari* on nitrogen mineralization and investigated the interaction between *P*. *liquidambari* and nitrogen, which in turn will be helpful to the farmer in reducing the required amount of soil N fertilizer. This change in N availability in maize soil will have significant implications for soil productivity and plant N utilization, especially in N-limited soils, and significantly reduce the required amount of soil N fertilizer. The effect of *P*. *liquidambari* on N mineralization in maize soil was investigated by treating it with four levels of N (urea) at rates of 0, 1.25, 2.5, and 3.75 g of nitrogen. N-mineralization was determined by the anaerobic incubation method. Were stored for 7 days in an incubator at a constant 37 C. A colorimetric microplate procedure was used for NH_4_^+^-N analysis. A significant increase in the available NH_4_^+^-N contents was reported in soil maize (*Zea mays* L.) inoculated with *P*. *liquidambari*, which increased by 80%. A significant increase in N-mineralization was observed under all N conditions. This work highlighted the importance of the fungal endophyte for soil N-mineralization with lower N input. Using this fungal agent will almost certainly help reduce fertilizer input.

## Introduction

Biological processes such as symbioses can increase agricultural sustainability by reducing dependency on non-renewable inputs such as inorganic synthetic fertilizers [1]. Many studies, for example, suggest that symbionts can improve host plant development and provide biotic and abiotic stress tolerance [2,3]. Fungal endophytes have been shown to promote soil nutrient collection, tolerance, and host plant development [4,5].

Maize (Zea mays L.) is a significant crop globally, widely used for feed and industrial raw materials. Maize ranks third in the world and is the first-largest crop in USA production [6]. Maize is a productive and multipurpose crop, responding to investments in research, breeding, and promotion. It has extremely high yields compared with most other U.S. crops, and it grows approximately anywhere in the United States, being especially successful in the Midwest and Great Plains. It is used mostly for food, fuel ethanol, animal feed, and high fructose corn syrup [7]. In the U.S. in 2019, according to the National Agricultural Statistics Service (NASS), farmers planted more maize than any other crop—nearly 91.7 million acres of maize [8].

Nitrogen fertilizer is one of the most important inputs to maize, and its use in maize is huge. Each year, over 5.6 million tons of nitrogen are applied to maize through chemical fertilizers in the United States, along with nearly a million tons of nitrogen from manure. Nonetheless, this costly input is subject to loss under different weather conditions [9]. N application rates have increased over the years. The U.S. corn crop production is falling along with supply estimates, according to the USDA. According to the Illinois Production Cost Report 2018, anhydrous ammonia prices averaged $522 per ton in Illinois. This $522 level is over $100 higher than September 2017 levels, when anhydrous ammonia prices averaged $401 per ton [10]. According to the USDA Report in June 2019, lowering corn yields by **10** bushels, making it the largest reduction in history, also reduced acres by **3** million [8].

Nitrogen mineralized from soil organic matter is the main source of crop nitrogen (N) uptake [11–13]. Nitrogen fertilizer application timing for maize production is one of the most expensive inputs for maize. Also, it is one of the most important things to ensure that maize crop growth is not nitrogen-limited and achieves the highest yield possible. Moreover, extreme weather in the Midwest may lead to economic risk, resulting in penalty costs due to the climate risk in the US. Wet soils result in deficient nitrogen when a second application is needed, which may result in lower yields [14].

Endophytes are microorganisms such as bacteria, fungi, and actinomycetes that inhabit intra- and intercellular plant tissues for all or part of their life cycle [15]. Endophytes can colonize the internal plant tissues of healthy leaves, petioles, stems, twigs, bark, roots, fruits, flowers, and seeds without causing any apparent harm or pathogenic infection to their host plants [16,17]. Endophytic fungi are an ecological and polyphyletic group of highly diverse fungi, mostly belonging to ascomycetes and anamorphic fungi [18].

Cross-host species inoculation of maize with *Phomopsis liquidambari* will be revealed if this endophyte forms a mutualistic symbiotic relationship with maize (*Zea mays* L.), promotes the growth and yield of maize, improves the N accumulation and N use efficiency of maize, and significantly reduces the required amount of soil N fertilizer [19]. Furthermore, a previous study reported that *Phomopsis liquidambari* stimulates the expression of several genes involved in N-uptake and the metabolism of rice seedlings [20]. These results indicate the beneficial effects of *Phomopsis liquidambari* on nitrogen use in rice plants, but in maize, they are largely unknown.

Fungal endophytes have been frequently reported to enhance host plant growth and improve plant nutrient uptake from soil [4,5,21]. For these reasons, our objective in this study was to determine if *Phomopsis liquidambari*, nitrogen levels, or both influence N-mineralization in maize soil and whether there is an interaction between *Phomopsis liquidambari* and nitrogen.

## Materials and methods

Maize seeds were planted on May 9, 2016. As Chaintreuil et al., (2000) [22] described, before planting, seeds were drenched in 96% ethanol for fifteen minutes, rinsed two times with sterile distilled water, then purified in 0.1% mercuric chloride (HgCl_2_) for 25 minutes, and rinsed six times in sterile distilled water. According to Feng et al., (2006) [23] and Yang et al., (2015) [24], we placed the sterilized seeds on a potato dextrose agar surface for five days at 28°C to make sure there were no pathogenic organisms.

In 100 mL of potato dextrose broth, we cultured *P*. *liquidambari* at 28°C for 3 days. Then, for four days at 28°C, submerged fermentation was carried out in flasks containing 500 mL of sterilized potato dextrose broth medium and 10% seed culture broth. In order to calculate the dry cell weight, 10 mL of culture broth has been used. Then, after cutting 3 g of dry fungal mycelia, we gave it two sterile distilled water washes before diluting it to a final volume of 200 mL. On the seeds that were germinating, the fungal suspension was applied. The seeds were randomly split into two groups and then transferred to Petri dishes after being sterilized. After that, the inoculated group (P+) was given 80 mL of the fungus for each dish, whereas the non-inoculated group (P-), which served as the control, received 80 mL of sterilized deionized water [24,25].

In 2016, soil for this study was collected from maize fields University of Benghazi at a depth of 0–15 cm in a bucket. Soil samples, yellowish-red loamy sand, were homogenized by hand, dried by air, and then added to plastic pots with a height of 35 cm and a diameter of 25 cm. The organic matter content was 2.05%. The soil pH was 7.24. The nutrient composition of the soil was: total N, 0.6% (Table 1).

**Table 1:** Chemical and physical properties of the experimental soil.

Soluble cations meq/100g		Soluble anions meq/100g	
***Ca* ^+2^**	0.22	***Cl* ^−^**	0.24
***Mg* ^+2^**	0.13	${HCO}_{3^{-}}$	0.2
***Na* ^+^**	0.04	${SO}_{4^{-}}$	0.08
***K* ^+^**	0.12		
Total Nitrogen (TN) (%)		0.6	
Total phosphorus (TP) ppm		16.48	
Organic matter (%)		2.05	
*Ph*		7.24	

Six replicated pots were used for each treatment. Four levels of nitrogen treatments were applied as urea at rates of 0 (zero), 1.25 (low nitrogen), 2.5 (moderate nitrogen), and 3.75 (high nitrogen) g of nitrogen (N) per pot. Replicate pots were grown and maintained for each treatment (zero N, low N, moderate N, and high N; P+ and P-; three plants per pot). Soil samples were collected during the V5 maize growth stage (5 collared leaves) on June 16, 2016. This growth stage coincides with the maximum rate of N mineralization [26]. The nitrogen mineralization was determined by the anaerobic incubation method [27].

### Procedure for anaerobic N-mineralization

This method was modified by Bundy and Meisinger (1994) [27], and we used it to determine initial and mineralizable N. We placed 1 g of stored sample in 4 replicate 16 x 125 mm glass test tubes and amended them with 13.5 mL of distilled H_2_O. We added a rubber stopper to one tube of each sample. The remaining tubes were capped with a rubber stopper and a metal jacket crimp due to gas production during sample incubation. We shook all tubes vigorously with a reciprocating shaker for 5 minutes. Then, the uncrimped stoppered tubes were amended with 7 mL of 4 M KCl in distilled H_2_O and incubated at 37°C for 7 days. After 7 days, we removed the crimped tubes from the incubator. Before the crimps were removed, we relieved gas pressure by venting it with a needle. Then 7 mL of 4M KCl were added to each tube. All samples were placed on a reciprocating shaker for 30 minutes, and 1 mL of the resulting suspended supernatant was placed in Eppendorf tubes and stored at -20 C until analysis of NH4^+^.

## Colorimetric analysis of NH_4_^+^

For the analysis of NH_4_^+^-N, a colorimetric microplate method was used [28,29]. This is a modification of the Berthelot indophenol blue reaction for the determination of ammonia [30]. We dispensed 20 μL of NH_4_^+^ standards (0 to 10 mg L^-1^) into the first two columns of a 96-well microplate (360 μL Greiner microplate). Then we dispensed 20 μL of supernatant from the anaerobic N-mineralization assay in successive, duplicate columns. Finally, in a fume hood, we dispensed 100 μL of Reagent I (a 1.0% phenol solution containing 0.020% sodium nitroprusside) and 100 μL of Reagent II (a 0.5% solution of sodium hydroxide containing 0.042% sodium hypochlorite) in succession to each microplate well. The plates were covered with film to minimize ammonia (NH_3_) volatilization during shaking and placed on a shaker for 30 minutes. The concentration of NH_4_^+^-N was determined at a wavelength of 630 nm on a microplate reader. Net NH_4_^+^-N production was calculated by subtracting the NH_4_^+^-N content of the initial samples from the incubated samples.

### Experiment design and statistical analysis

This project was designed as a completely random design. We were interested in the effects of two *Phomopsis liquidambari* (infected P+ and uninfected P-) and four levels of N on N-mineralization. We perform six replicates of a 2× 4 factorial treatment design. All data were subjected to analysis of variance (2-way ANOVA) with Microsoft SAS 9.4 (SAS Institute, 2012). The significant differences were separated by Tukey at P < 0.05 to provide a 95% confidence limit for the separation of treatment effects.

## Results

To evaluate the effect of the endophytic fungal and low nitrogen (urea) input on N-mineralization, the analysis of variance (two-way ANOVA) showed a highly significant interaction between *Phomopsis* and levels of nitrogen (F_3, 40_ = 7.97, P = 0.0003). There was a significant effect of the soil inoculated with *Phomopsis liquidambari* on N-mineralization (F_1, 40_ = 96.98; P < 0.0001) and N fertilizer application rates (F_3, 40_ = 61.64; P < 0.0001).

**Fig 1:**
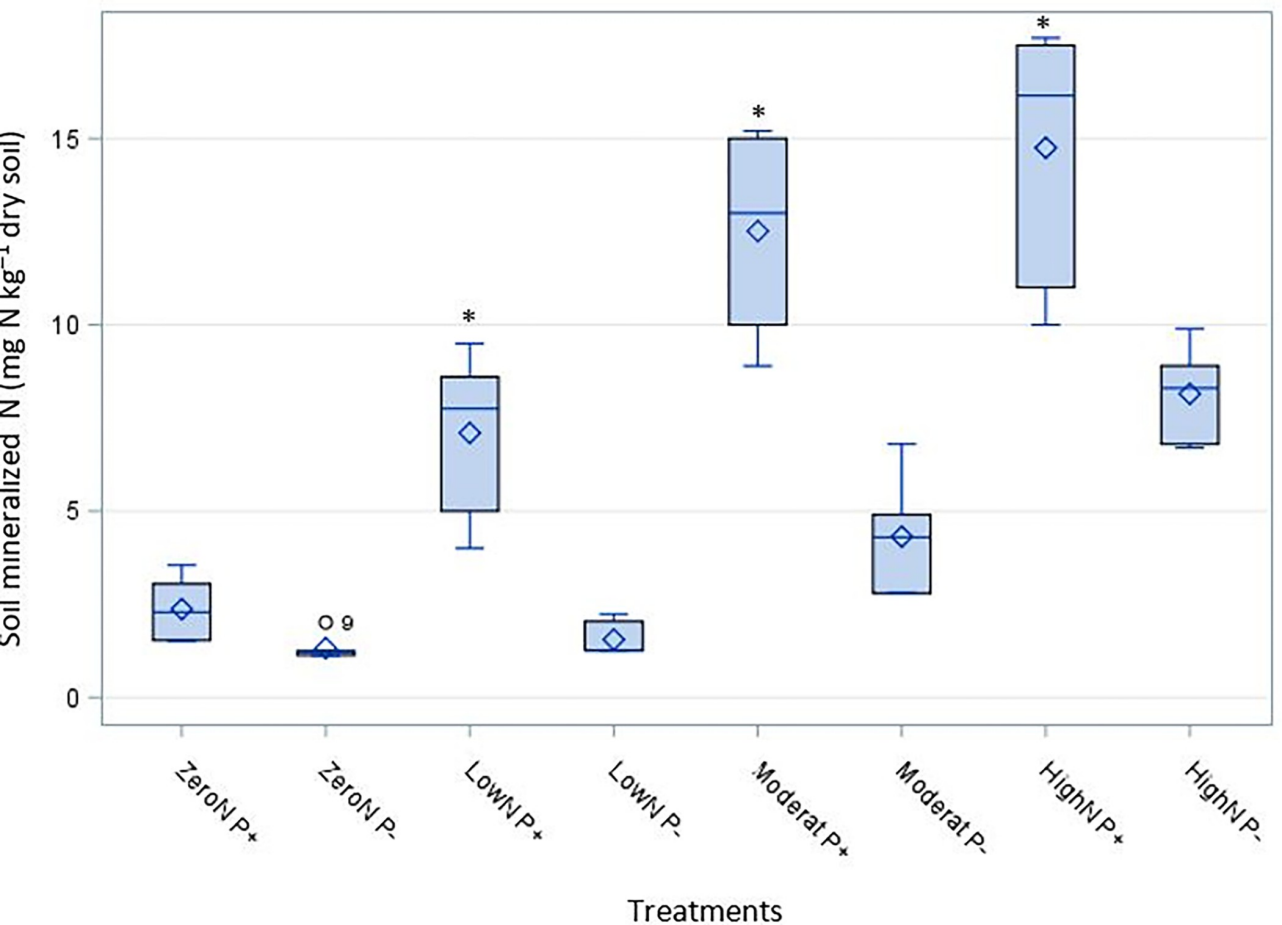
Effects of *P*. *liquidambari* & N- Levels on N-mineralization in maize soil. Four levels of nitrogen treatments were applied at rates of 0 (zero), 1.25g (low nitrogen), 2.5g (Moderate nitrogen), and 3.75g (high nitrogen) and (P+) inoculated with *Phomopsis liquidambari* (P-) non-inoculated with *Phomopsis liquidambari*. Boxplots represented by the asterisk(*) are statistically different by Tukey’s honest significance test (P<0.05).

Across all N rates, N-mineralization in maize soil was significantly increased in treatments inoculated with *P*. *liquidambari* than in non-inoculated treatments, where low, moderate, and high N rates were (p < 0.0002, p < 0.0001, and p < 0.0001, respectively) (Fig 1).

At a low N rate, N-mineralization in the inoculated maize soil was 7.10 ± 2.14 mg kg^-1^; a moderate N rate was 12.57 ± 2.59 mg kg^-1^; and N mineralization was greater in soil treated with *P*. *liquidambari* at a high N rate. It was 14.8± 3.48 mg kg^-1^ greater than the non-inoculated, regardless of N rate. However, there was a non-significant effect on N-mineralization in either inoculated or non-inoculated with *P*. *liquidambari* under zero N (Table 2).

**Table 2:** Effects of *P*. *liquidambari* and N- Levels on N-mineralization in maize soil.

NH_4_+—N mg kg^-1^ Nitrogen Levels				
*P*. *liquidambari*	Zero N	Low N	Moderate N	High N
P-	1.32 ± 0.34	1.55 ± 0.46	4.32 ± 1.51	8.15 ± 1.26*
P+	2.37 ± 0.83	7.10 ± 2.14*	12.57 ± 2.59*	14.8 ± 3.48*

Means ± Standard deviations with * are significantly different at P ≤ 0.05 (Tukey’s HSD). Four levels of nitrogen treatments were applied at rates of 0 (zero), 1.25g (low nitrogen), 2.5g (Moderate nitrogen), and 3.75g (high nitrogen) and (P+) inoculated with *Phomopsis liquidambari* (P-) non-inoculated with *Phomopsis liquidambari*.

On average, the data shown in Fig 1 suggests an increase in N-mineralization with increased nitrogen levels. At a high N rate, N-mineralization in maize soil non-inoculated *P*. *liquidambari* was significantly affected (8.15 ± 1.26 mg kg^-1^).

## Discussion

Our results indicate that *Phomopsis liquidambari* increases N-mineralization even when nitrogen is low. The sum of squares values, which are a measure of the amount of variation explained by each effect, suggested that *P*. *liquidambari* had the most effect on N-mineralization, followed by nitrogen and the nitrogen- *P*. *liquidambari* interaction. *P*. *liquidambari* significantly increases the potential for N-mineralization under all N conditions.

In regards to N mineralization rates, we found that the N mineralization rates were significantly higher in maize soil inoculated with *P*. *liquidambari* than in non-inoculated. The fungal endophyte shows a similar role in improving the available N levels in rice soil; their studies have demonstrated that *P*. *liquidambari* has beneficial effects on N utilization in rice [20]. A possible explanation for increasing N mineralization in the inoculated maize soil could be due to the change in microbial mineralization, community structure, and abundance caused by endophytic colonization. Endophyte colonization might affect the N-related microbial composition and cause large changes in the quantitative and qualitative exudation parameters, and an increase in root exudation to the surrounding soil could have a useful result on the processes of N-transforming [31].

Furthermore, N mineralization can be improved by fastening soil organic matter degradation and microbial metabolism, which can be enhanced by a higher soil nutrient supply. Also, N mineralization could be explained by the abiotic and biotic factors linked with the improved environmental conditions, such as increased soil microbial biomass, which significantly enhanced the metabolism of microorganisms [16,32], ultimately stimulating the activities of soil microbes [33].

Increased N-mineralization has been observed with increased N fertilizer (urea) rates in our study. Binh and Shima (2018) [34] found a similar result of rapid N immobilization in soils treated with N sources. Their results indicated that the net N mineralization was greater in soil treated with soil incorporated with bamboo stems or rice straw. Also, Zhang et al., (2015) [35] results suggest that N fertilization as urea increased N mineralization, nitrification, inorganic N flux, and NH_3_ volatilization while reducing the economic efficiency of added N.

## Conclusion

Understanding how endophytic fungal *P*. *liquidambari* affects soil N mineralization will improve N fertilizer management, resulting in improved soil productivity and reduced unfavorable environmental results. In our study, *Phomopsis* increased N-mineralization even when nitrogen was low; however, N mineralization was greater in soil treated with endophytic fungal *P*. *liquidambari* at high N fertilizer rates. Our present study suggests that *Phomopsis* significantly increases the potential N-mineralization under all N conditions. Further research into how this fungus impacts N mineralization and microbial communities is needed to better understand the mechanism of interaction between *Phomopsis* and levels of nitrogen and increased N-mineralization.

## Supporting information

S1 Data (XLSX)
